# An evaluation of pharmacists’ general attitudes, knowledge, and phobias regarding medications that include corticosteroids: a cross-sectional study

**DOI:** 10.1186/s40780-024-00329-x

**Published:** 2024-02-01

**Authors:** Muna Barakat, Samar Thiab, Rana Abu Farha, Anas O. Alshweiki, Roa’a Thaher, Asem Alsughaier, Diana Malaeb

**Affiliations:** 1 Department of Clinical Pharmacy and Therapeutics, School of Pharmacy, Applied Science Private University, Amman, Jordan; 2 Department of Pharmaceutical Chemistry and Pharmacognosy, Faculty of Pharmacy, Applied Science Private University, Amman, 11931 Jordan; 3 Department of Pharmaceutical Sciences and Pharmaceutics, Faculty of Pharmacy, Applied Science Private University, Amman, Jordan; 4 Medical Intern, Hamad Medical Corporation, Hamad, Qatar; 5 Al-Balqa Applied University, Al-Salt, Jordan; 6 College of Pharmacy, Gulf Medical University, P.O. Box 4184, Ajman, United Arab Emirates

## Abstract

**Background:**

Corticosteroid-containing medications are widely accessible in various forms, including topical, injectable, and inhaled formulations. Due to uncertain safety profiles, healthcare providers, including pharmacists, often express apprehension when dispensing these drugs. This cross-sectional study assesses the knowledge, attitudes, and phobia of Jordanian pharmacists regarding corticosteroid-containing medications.

**Methods:**

Conducted through a self-administered online questionnaire.

**Results:**

the study reveals that dermatological conditions and respiratory disorders are the primary indications for prescribing corticosteroid-containing drugs. The most reported side effects among pharmacists’ patients include increased appetite, diabetes, and skin thinning. Pharmacists generally exhibit acceptable knowledge, with a median score of 9.0 out of 11.0 (IQR = 3.0). Over two-thirds of pharmacists (69.9%) achieve a high knowledge score (Bloom’s cut-off point ≥ 8.8). However, only 55.7% are aware that corticosteroids may induce mood changes. High phobia scores, particularly concerning increased blood pressure and osteoporosis risks, indicate pharmacist reservations in corticosteroid dispensing. Interestingly, pharmacists in rural areas display lower knowledge scores, while those working outside community pharmacies exhibit lower phobia scores compared to their counterparts in urban areas and community pharmacies, respectively.

**Conclusion:**

Despite generally good knowledge levels, the study underscores high phobia scores among Jordanian pharmacists regarding corticosteroid dispensing, particularly due to concerns about blood pressure elevation and osteoporosis risks. This suggests a potential need for targeted educational interventions and support systems to enhance pharmacist confidence and optimize corticosteroid usage while minimizing associated risks.

**Supplementary Information:**

The online version contains supplementary material available at 10.1186/s40780-024-00329-x.

## Introduction

Corticosteroid-containing drugs are considered one of the main therapeutic options for many health conditions. Although various specialties in the medical sector widely use them, caution and care must be heavily applied to avoid possible side effects or toxicities [1]. Corticosteroids (CS) are available in inhaled dosage forms, orally ingested dosage forms, ophthalmic preparations, and parenteral and topical preparations. This variation in formulations and dosage forms facilitates common use by healthcare providers in regular and emergency settings [2].

The safety profile of corticosteroids is uncertain, and healthcare providers tend to develop hesitance or phobia toward the corticosteroid’s prescription. Exaggerated concerns, doubt, phobia, and resistance to receive such drugs can be called “corticophobia” [3]. This issue is directly linked to reduced adherence to treatment plans by patients. Several scientific tools are available to assess the severity of the impact of corticophobia on patients’ adherence to treatment plans and other aspects related to corticosteroids use by healthcare providers [4]. In addition, due to the unique nature of corticosteroids, continuous evaluation of the existing knowledge of corticosteroids among healthcare providers and the possible ways to improve this knowledge is a priority in research. Accordingly, this could improve the safe prescribing and dispensing of these drugs, or identify existing barriers to successful treatment plans based on corticosteroids [5, 6].

Furthermore, the perception of patients toward corticosteroid use is significantly different from healthcare providers’ perceptions. Patients tend to focus more on corticosteroid side effects as their main inconvenience, whereas physicians or pharmacists look more for the therapeutic impact and how it outweighs possible shortcomings [7, 8].

Corticosteroids use and COVID-19 have been heavily researched in the past few years due to the use of such drugs in COVID-19 management guidelines. Their role in treating symptoms, and reducing severe complications, such as respiratory tract inflammation among other specific roles for these drugs was monitored and recorded by various healthcare providers each in their unique setting [9]. Because of that the efficacy and safety of corticosteroids use in clinical settings are important to determine which steps should be considered to be implemented next to achieve better outcomes and avoid related side effects [10, 11].

In order to evaluate current practices and perceptions, and to measure how close are these behaviors to corticosteroid guidelines, research efforts were put to assess perceptions, measure knowledge, and determine the actual level of corticophobia among healthcare providers [12]. In Jordan, healthcare providers tend to have certain preferences or phobias toward prescribing and dispensing corticosteroids to patients in need. These preferences or phobias arise from the direct effect of multiple factors including economic, social, commercial, and medical factors which were investigated in this research. This study aims to investigate the pharmacists’ general attitudes, knowledge, and phobias regarding medications that include corticosteroids.

## Methods

This observational cross-sectional study was conducted in Jordan between November 2022 and February 2023. Participants were recruited using a convenience, snowball sampling technique, as the questionnaire was distributed through these platforms (WhatsApp, Facebook, and LinkedIn) and completed voluntarily and anonymously. Noteworthy, WhatsApp is a secure messaging app for text, voice, and media communication [13, 14], Facebook is a leading social networking platform for sharing updates, photos, and videos with friends [15], and LinkedIn is a professional networking site for career development, allowing users to showcase profiles, connect with colleagues, and explore job opportunities in a business-oriented environment [16]. The questionnaire was made to be a self-administered tool. Any pharmacist who practices in Jordan and is willing to answer the questionnaire was eligible to participate. The participants were given a written consent statement at the start of the survey: “Your participation in completing this questionnaire is highly appreciated.” The participants accepted the consent if they were willing to continue with the survey. They did not answer the survey questions after selecting “disagree to participate” if that was the case. When potential participants filled out the survey, it was assumed that they gave their informed consent to take part in the research. Institutional Review Board (or Ethics Committee) of the Faculty of Pharmacy, Applied Science Private University, Amman, Jordan, has obtained ethical approval (approval number:2022-PHA-24).

### Study tool

The study questionnaire was developed after a review of pertinent validated surveys found in the literature [12, 17–19] and adherence to the fundamental principles of successful survey design [20]. Through the development of the tool, a pool of questions important to the objectives of the study was added. The questionnaire was divided into four categories with multiple choice questions: sociodemographic (5 questions), CS dispensing experience (4 questions), CS knowledge (11 questions), and CS dispensing phobias and desires (10 questions).

The study tool written in English was evaluated for content validity by six academics. To ensure that all items in the questionnaire were clear, 15 pharmacists participated in a pilot study. Based on their comments, linguistic modifications were made when needed. The pilot replies were not taken into consideration in the final analyses. The questionnaire was then electronically circulated in its final form.

The responses to the 11 knowledge assessment items were used to compute the total knowledge score. Based on the sum of the individual item scores (1 for a right answer), the general knowledge score has a range of 0 to 11. Then, we categorized the knowledge score into high and low using the 80% Bloom’s cut-off point [21]. So, high knowledge score refers to participants who scored 8.8–11.0 (≥ 80%), and low knowledge score refers to those who scored 0-8.7 (< 80%). Additionally, a Likert scale with a maximum of five points was employed to record the replies about the terror score (strongly disagree = 1, disagree = 2, neutral = 3, agree = 4, and strongly agree = 5). The reliability and internal consistency of the scale were validated with a Cronbach’s alpha of 0.85.

The responses to the 5-point Likert-type scale were assessed, and the mean and standard deviation of the phobia score were determined for each item and sample category (physicians and pharmacists). The score results have been divided into three rankings [22], where low scores lie between 1.00 and 1.66, moderate scores between 1.67 and 3.32, and high scores between 3.33 and 5.00.

### Statistical analyses

Data were extracted from Google Forms as an Excel sheet and were then exported to Statistical Package for Social Sciences version 25.0 (IBM SPSS Statistics for Windows, version 25 (IBM Corp., Armonk, N.Y., USA) for statistical analysis. The descriptive analyses were conducted using median and Interquartile Range (IQR) for continuous variables, and frequency and percentages for categorical variables.

Independent factors that may affect the participants’ knowledge about CS and participants’ phobias towards CS were investigated using univariate linear regression analysis. Then variables, that were found to be significant on a single predictor level (*p* < 0.25) were entered into multiple linear regression analyses. Variables were selected after checking their independence, where tolerance values > 0.1 and Variance Inflation Factor (VIF) values < 10 were checked to indicate the absence of multicollinearity between the independent variables in regression analysis. None of the included variables showed multicollinearity, thus, none was eliminated. Statistical significance was considered at *p* ≤ 0.05.

## Results

### Sociodemographic characteristics of the study participants

A total of 184 responses have been received, ten of them declined the participation and 174 agreed to take part in this study. The response rate was 95%, and the complete responses (*n* = 174) were included in the final analysis. The median age of them was 27.0 years (IQR = 12.3). More than half of them were males (*n* = 90, 51.7%), and most of them were residing in urban areas (*n* = 166, 95.4%). The majority of the recruited pharmacists were working in the center of Jordan (*n* = 152, 87.4%). Regarding their site of work, around two-thirds of the pharmacists were working in community pharmacies (*n* = 110, 63.2%), while 6.3% of them were working in hospital settings (*n* = 11). Details about sociodemographic characteristics are presented in Table 1.

**Table 1:** Socio-demographic characteristics of the study respondent (*n* = 174)

Parameter	Median (IQR)	n (%)
Age (years) (median (IQR))	27.0 (12.3)	
Gender		
Males		90 (51.7)
Females		84 (48.3)
Residence area		
Urban		166 (95.4)
Rural		8 (4.6)
Pharmacists site of work		
Community pharmacy		110 (63.2)
Hospital pharmacy		11 (6.3)
Pharmaceutical company		11 (6.3)
Medical representative		10 (5.7)
Others		32 (18.4)
Location of work		
Center of Jordan		152 (87.4)
North of Jordan		18 (10.3)
South of Jordan		4 (2.3)

IQR: interquartile range

### Pharmacists’ experience with dispensing corticosteroids

Regarding pharmacists’ experience in dispensing CS (Table 2), around half of them reported to have experience dispensing CS (*n* = 91. 52.3%). Among those who had dispensed CS, the most common dosage form dispensed by the pharmacists was topical (*n* = 84/91, 92.3%) followed by injectable dosage forms (*n* = 75/91, 82.4%). Moreover, the main indications for dispensing CS were dermatological diseases (*n* = 84/91, 92.3%) followed by respiratory diseases (*n* = 79/91, 86.8%). In addition, the most common side effects of CS the pharmacists experienced with their patients were increased appetite (*n* = 62/91, 68.1%), followed by diabetes (*n* = 58/91, 63.7%), and thinned skin (*n* = 48/91, 52.7%).

**Table 2:** Pharmacists experience with corticosteroids (*n* = 174)

Questions	n (%)
Have you ever dispensed corticosteroids for any reason?	
Yes	91 (52.3)
No	65 (37.4)
Maybe	18 (10.3)
The dosage form of corticosteroids you have dispensed$	
Topical (e.g., cream or ointment)	84 (92.3)
Inhaler or nebulizer	65 (71.4)
Tablets	62 (68.1)
Injection	75 (82.4)
Drops (e.g., eye drops)	65 (71.4)
The indication for corticosteroid you have dispense$	
Respiratory disease (e.g., Asthma, COPD)	79 (86.8)
COVID-19	52 (57.1)
Dermatological disease (e.g., eczema)	84 (92.3)
Joint or rheumatological diseases	59 (64.8)
GIT immunological diseases (e.g., Crohn’s disease)	38 (41.8)
Systemic immunological disorders (e.g., Multiple Sclerosis)	42 (4.2)
Did you ever experience any of the following side effects with your patients? $	
Increased appetite – potentially leading to weight gain.	62 (68.1)
Acne	27 (29.7)
Thinned skin that bruises easily	48 (52.7)
Increased risk of infections	35 (38.5)
Mood changes, mood swings, and depression	42 (46.2)
Diabetes	58 (63.7)
High blood pressure	43 (47.3)
Osteoporosis (weak and brittle bones)	43 (47.3)

$ Percentages calculated out of 91, hence, more than one answer was allowed

### Pharmacists’ knowledge about corticosteroids

In general, pharmacists had a median score of 9.0 out of 11.0 (IQR = 3.0) (Table 3). More than two-thirds of the pharmacists (*n* = 241, 69.9%) had a high knowledge score (Bloom’s cut-off point ≥ 8.8). Pharmacists were knowledgeable to recognize that CS causes weight gain (*n* = 167, 96.0%), identify that prolonged steroid treatment at high doses can cause problems in some people (*n* = 159, 91.4%), and that CS are considered as anti-inflammatory medicine (*n* = 152, 87.4%). Surprisingly, only 55.7% of the pharmacists knew that CS causes mood changes in patients (*n* = 97. 55.7%), and around 62% of them recognized that CS can cause high blood glucose (*n* = 108, 62.1%).

**Table 3:** Pharmacists’ knowledge about corticosteroids medications (*n* = 174)

Statement	Correct answer	Percentage of patients correctly answered n (%)
Corticosteroids, often known as steroids, are anti-inflammatory medicine.	True	152 (87.4)
Corticosteroids are man-made hormones normally produced by the adrenal glands.	True	137 (78.7)
Corticosteroids are mainly used to induce inflammation and suppress the immune system.	False	140 (80.5)
Corticosteroids are used to treat various health conditions (e.g., asthma, eczema, COVID-19 etc.)	True	126 (72.4)
Prolonged steroid treatment even at low doses – particularly with steroid tablets – can cause problems in some people.	False	159 (91.4)
The used dose needs to be reduced slowly over few weeks or months before stopping corticosteroids if have been taking them long time.	True	156 (89.7)
Corticosteroids can cause weight gain	True	167 (96.0)
Corticosteroids can cause skin thinking that bruises easily	False	142 (81.6)
Corticosteroids can cause increased risk of infections	True	137 (78.7)
Corticosteroids can’t cause mood changes	False	97 (55.7)
Corticosteroids can cause high blood glucose	True	108 (62.1)
Knowledge score (out of 11), median (IQR)	9.0 (3.0)	

### Pharmacists’ phobias toward corticosteroids dispensing

Pharmacists showed a high phobia score toward corticosteroid dispensing (median score of 3.9, IQR = 0.9). Pharmacists were mainly afraid of dispensing CS due to the risk of increasing blood pressure among patients and the risk of osteoporosis (*n* = 139, 79.9% for both). Also. Pharmacists were afraid of the risk of weight gain among their patients (*n* = 130, 74.7%), Table 4.

**Table 4:** Fears toward corticosteroids dispensing among study participants (*n* = 174)

I am afraid to dispense corticosteroids due to………….	Percent agreed/strongly agreed	Median fear score (IQR)
Risk of weight gain among my patients	130 (74.7)	4.0 (1.0)
Risk of hyperglycemia among my patients	114 (65.5)	4.0 (2.0)
Risk of increased blood pressure among my patients	139 (79.9)	4.0 (2.0)
Risk of osteoporosis among my patients	139 (79.9)	4.0 (1.0)
Risk of patient’s addiction to this medication and not being able to live without it	99 (56.9)	3.0 (2.0)
Risk of developing adrenal insufficiency after stopping the corticosteroids among my patients	113 (64.9)	4.0 (2.0)
Dosage form (oral and injectable) more than the topical products	118 (67.8)	4.0 (2.0)
Risk of patient’s-social refusal if they know about the corticosteroids use	106 (60.9)	4.0 (1.0)
Risk of depression of mood swings among my patients	77 (44.3)	3.0 (1.0)
Risk of any unknown/untreatable side effects among my patients	116 (66.7)	4.0 (1.0)
*Fears score, median (IQR)		**3.9 (0.9)**

*(1.00-1.66): Low score on the Likert scale, (1.67–3.32): Moderate score on the Likert scale, (3.33-5.00): High score on the Likert scale

### Predictors of factors affecting pharmacists’ knowledge about corticosteroids

Regression analysis for factors influencing pharmacists’ knowledge about corticosteroids (Table 5) showed that the pharmacists living in rural areas had a 0.206 lower knowledge score compared to those living in urban areas (*p* = 0.006). The overall regression analysis was found to be significant, *p* = 0.003. The model accounted for 6.4% of the variance in predicting pharmacists’ knowledge score (R2 = 0.064).

**Table 5:** Assessment of factors associated with participants’ knowledge about corticosteroids

Parameter	Knowledge score			
	Beta	P-value#	Beta	P-value$
Age (years) median (IQR)	0.147	0.053^	0.144	0.053
Gender				
Males	Reference			
Females	-0.078	0.305	---	---
Residence area				
Urban	Reference			
Rural	-0.208	0.006^	-0.206	0.006*
Pharmacists site of work	Reference			
Community pharmacy	0.083	0.279	---	---
Others				
Location of work				
Center of Jordan	Reference			
Others	0.055	0.470	---	---

# using simple linear regression, $ using multiple linear regression, ^ eligible for entry in multiple linear regression, * significant at 0.05 significance level

### Predictors of factors affecting pharmacists’ phobias towards corticosteroids

Moreover, regression analysis was used to evaluate factors influencing pharmacists’ phobias towards corticosteroids (Table 6), and results showed that pharmacists working in sites other than the community pharmacies had a 0.151 lower phobia score compared to those working in community pharmacies (*p* = 0.049). The overall regression analysis was found to be significant, *p* = 0.016. The model accounted for 5.9% of the variance in predicting pharmacists’ phobia score (R2 = 0.059).

**Table 6:** Assessment of factors associated with participants’ fears from corticosteroids

Parameter	Fear score			
	Beta	P-value#	Beta	P-value$
Age (years) median (IQR)	0.052	0.499	---	---
Gender				
Males	Reference			
Females	0.116	0.128^	0.093	0.222
Residence area				
Urban	Reference			
Rural	-0.073	0.336	---	---
Pharmacists site of work				
Community pharmacy	Reference			
Others	-0.185	0.014^	-0.151	0.049*
Location of work				
Center of Jordan	Reference			
Others	-0.153	0.044^	-0.133	0.079

# using simple linear regression, $ using multiple linear regression, ^ eligible for entry in multiple linear regression, * significant at 0.05 significance level

## Discussion

This is the first study in Jordan to assess pharmacists’ knowledge, experience, and phobia toward dispensing CS to patients. Around half of the pharmacists who participated in this study had dispensed CS medications before. Several dosage forms of CS are available as over-the-counter (OTC) medications, including creams, lotions, ointments, and nasal sprays [23, 24]. As most of the corticosteroids that the pharmacists can dispense are topical, they were found to be the most dispensed dosage forms to the patients. Moreover, because inhaled CS is known as an effective medications to control asthma and treat allergic rhinitis [25, 26], inhaled CS came in second place as the most dispensed dosage form. Based on the literature, the most common adverse effects of CS include hyperglycemia and diabetes, hypertension, skin thinning and fragility due to atrophy, fractures due to osteoporosis or osteonecrosis, adrenal suppression, myopathy, psychiatric disturbances, immunosuppression as well as cardiovascular and gastrointestinal adverse effects [27–29]. Additionally, prednisolone, which is a well-known CS was found to act as an appetite stimulant in patients with cancer [30]. Pharmacists in this study noted increased appetite, diabetes, and thinned skin as the most common adverse effects among their patients, which is consistent with the reported side effects of CS in the literature.

Pharmacists showed an overall good knowledge of corticosteroids’ indications, adverse effects, and the need for dose tapering before stopping the medication. Pharmacists were shown to have good pharmacological knowledge, as their study focuses on pharmacology and pharmaceutical care [31, 32]. They are expected to provide counseling about the duration, frequency, and quantity of the corticosteroid to be used as prescribed by the doctors and also educate patients about the use of corticosteroids. Previous studies conducted in Jordan found that nearly all pharmacists provide basic information about medication use, including advice to patients regarding the medications’ proper indication, dosage regimen, and any possible food-drug interactions [12, 33]. Nonetheless, only around 55% of the participants were aware that CS can cause mood and psychological disturbances. Psychological illness is still surrounded by social stigma in Jordan [34–36], which can have a significant impact on various aspects of society, including healthcare and knowledge dissemination among healthcare professionals, due to the lack of awareness and limited knowledge on the subject [37].

Pharmacists showed a high phobia score toward CS as they were worried about the increase in blood pressure among the patients, as well as, the risk of weight gain and developing osteoporosis. The reason for pharmacists’ phobia can be also explained due to their good knowledge of the side effects of CS because of the nature of their study, which focuses on the mechanism of action of drugs [31, 32, 38]. On the other hand, a study regarding the management of corticophobia was conducted in Jordan in 2013, reported that the intervention of clinical pharmacists has a significant impact on lowering patients’ fear of corticosteroids, thus improving their compliance with corticosteroids treatment regimens [17]. This was also concluded in a review, where pharmacists were found to be essential and accessible healthcare providers who have the potential to significantly improve medication adherence [39].

Although pharmacists participating in this study showed acceptable knowledge about CS, it was found that pharmacists living in urban areas have better knowledge than those living in rural areas. Factors that might potentially affect the perception of lower knowledge scores among pharmacists in rural areas include the practice settings as there are fewer healthcare professionals in the area, limited access to educational resources, as most universities [40], research centers, and training workshops are available in urban areas, which impact their exposure to the latest developments and advancements. Finally, urban areas generally provide more opportunities for networking and collaboration among healthcare professionals, including pharmacists, compared to rural areas and this can impact the exposure and exchange of information. The results also showed that community pharmacists had higher phobias than others working in different settings. This can be due to the fact that pharmacists working in community pharmacies often deal with a high volume of patients typically directly, as they were shown in a study conducted in Jordan to be the most trusted, accessible, and affordable healthcare providers in the country [41], so a high number of people seek their help, and this can sometimes expose them to complex medication-related issues. In addition, they deal with more frequent interruptions [42], and time constraints compared to pharmacists in other settings, which may create a more stressful environment, potentially leading to higher phobia levels.

## Study limitations

Several limitations apply to this study. First, the small sample size and the sole representation of pharmacists in Jordan would make it difficult to generalize our findings. One more significant problem is selection bias connected to the snowball technique used for data collecting. Instead of having participants meet in person, surveys conducted online could impair the validity and authenticity of study data. Moreover, our data collection process did not include the quantification of the number of dispensing cases of CS by the pharmacists, the reported side effects, and the pharmacist’s role to manage and counseling the patients. Finally, there were a large number of pharmacists who are not working in clinical settings, which makes the interpretation of the results very difficult, so, a similar study could be conducted in the future to understand their perspectives and experience with CS-containing drugs. As well, future qualitative studies that employ theoretical frameworks for data collection and analysis will give in-depth insights into the viewpoints and experiences of the healthcare industry.

## Conclusion

The findings of the current study provide important light on the knowledge and practice among pharmacists in Jordan. Our data show that pharmacists have a good knowledge level about corticosteroids, however, they showed a high phobia score toward CS as they phobia from the side effects. Based on these results, pharmacists can offer complete, patient-centered care that maximizes the proper use of CS while lowering their hazards.

### Electronic supplementary material

Below is the link to the electronic supplementary material.

**Supplementary Material 1:** Supplementary Material-Study Questionnaire

## Data Availability

Not applicable.

## Abbreviations

CS: Corticosteroids
COVID-19: Coronavirus disease of 2019
IQR: Interquartile Range

## References

[1] Michelle Kapugi KC, Corticosteroids Pharmacology CE. 2019;38(5):4. 10.1097/NOR.0000000000000595 31568125

[2] Nazir A, Masih M, Iqbal M (2021). Formulation, optimization, qualitative and quantitative analysis of new dosage form of corticosteroid. Future J Pharm Sci.

[3] Bos B (2019). Corticosteroid phobia (corticophobia) in parents of young children with atopic dermatitis and their health care providers. Pediatr Dermatol.

[4] Mueller SM et al. *Assessment of corticophobia as an indicator of non-adherence to topical corticosteroids: a pilot study*. J Dermatological Treat, 2016: p. 9. 10.1080/09546634.2016.1201189 27396480

[5] Duyvendak M (2011). Doctors’ beliefs and knowledge on corticosteroid-induced osteoporosis: identifying barriers to improve prevention. J Clin Pharm Ther.

[6] Kang MJ (2020). Community pharmacists’ knowledge, perceptions, and practices about topical corticosteroid counseling: a real-world cross-sectional survey and focus group discussions in Korea. PLoS ONE.

[7] Jaffuel D et al. *Perception of oral corticosteroids in adult patients with asthma in France*. J Asthma. 2020;13. 10.1080/02770903.2020.1748048 32285714

[8] Oluwaseyi I. *The Perceived Accompanying Dangers Of Dexamethasone (A Corticosteroid) Use In Covid-19 Management*. 2020 6/2020; Available from: https://www.researchgate.net/publication/342282696_THE_PERCEIVED_ACCOMPANYING_DANGERS_OF_DEXAMETHASONE_A_CORTICOSTEROID_USE_IN_COVID-19_MANAGEMENT.

[9] Annane D (2021). Corticosteroids for COVID-19. J Intensive Med.

[10] Annane D, Bollaert BE, Briegel PE, Keh J, Kupfer D, Pirracchio Y, Rochwerg R. B, *Corticosteroids for treating sepsis in children and adults* 2019: p. 163. 10.1002/14651858.CD002243.pub4 PMC6953403 31808551

[11] Russell B et al. COVID-19 and treatment with NSAIDs and corticosteroids: should we be limiting their use in the clinical setting? Ecancermedicalscience. 2020;14. 10.3332/ecancer.2020.1023 PMC7105332 32256706

[12] Barakat M (2023). Assessment of Knowledge, Perception, Experience and Phobia toward corticosteroids Use among the General Public in the era of COVID-19: a multinational study. Healthcare.

[13] Dahdal S (2020). Using the WhatsApp social media application for active learning. J Educational Technol Syst.

[14] Mayfield A. *What is social media* 2008.

[15] Habes M (2018). The relationship between social media and academic performance: Facebook perspective. Int J Inf Technol Lang Stud.

[16] Utz S (2016). Is LinkedIn making you more successful? The informational benefits derived from public social media. New Media Society.

[17] Ahmad DS, Wazaify MM. Albsoul-Younes, *the role of the clinical pharmacist in the identification and management of corticophobia–an interventional study*. Trop J Pharm Res. 2014;13(3):445–53.

[18] Choi E, Chandran NS, Tan C (2020). Corticosteroid phobia: a questionnaire study using TOPICOP score. Singapore Med J.

[19] El Hachem M (2017). Topical corticosteroid phobia in parents of pediatric patients with atopic dermatitis: a multicentre survey. Ital J Pediatr.

[20] Boynton PM (2004). Administering, analysing, and reporting your questionnaire. BMJ.

[21] Kaliyaperumal K (2004). Guideline for conducting a knowledge, attitude and practice (KAP) study. AECS Illumination.

[22] Abazid H et al. Public knowledge, beliefs, psychological responses, and behavioural changes during the outbreak of COVID-19 in the Middle East. Pharm Pract. 2021;19(2). 10.18549/PharmPract.2021.2.2306 PMC8216710 34221198

[23] Service. NH. *Topical corticosteroids*. 2020 [cited 2023 11/5]; Available from: https://www.nhs.uk/conditions/topical-steroids/.

[24] American Academy of Allergy, A.I. Over-The-Counter Allergy Nasal Steroid Sprays - What Does It Mean For Patients? 2020 [cited 2023 11/5]; Available from: https://www.aaaai.org/tools-for-the-public/conditions-library/allergies/triamcinolone-nasal-spray.

[25] Barnes PJ (2010). Inhaled corticosteroids. Pharmaceuticals.

[26] Lohia S, Schlosser R, Soler Z (2013). Impact of intranasal corticosteroids on asthma outcomes in allergic rhinitis: a meta-analysis. Allergy.

[27] Hodgens A, Sharman T. *Corticosteroids*, in *StatPearls [Internet]*. 2022, StatPearls Publishing. 32119499

[28] Buchman AL (2001). Side effects of corticosteroid therapy. J Clin Gastroenterol.

[29] Peppa M, Krania M, Raptis SA. Hypertension and other morbidities with Cushing’s syndrome associated with corticosteroids: a review. Integr Blood Press Control. 2011;7–16. 10.2147/IBPC.S9486 PMC3172078 21949634

[30] Willox JC (1984). Prednisolone as an appetite stimulant in patients with cancer. Br Med J (Clinical Res ed).

[31] Keijsers CJ (2014). A comparison of medical and pharmacy students’ knowledge and skills of pharmacology and pharmacotherapy. Br J Clin Pharmacol.

[32] Keijsers CJ (2015). Pharmacists’ and general practitioners’ pharmacology knowledge and pharmacotherapy skills. J Clin Pharmacol.

[33] Elayeh E (2017). Practice of pharmaceutical care in community pharmacies in Jordan. Trop J Pharm Res.

[34] Rayan A, Jaradat A (2016). Stigma of mental illness and attitudes toward psychological help-seeking in Jordanian university students. Res Psychol Behav Sci.

[35] Al Ali NM (2017). Factors affecting help-seeking attitudes regarding mental health services among attendance of primary health care centers in Jordan. Int J Mental Health.

[36] Hasan AA-H, Musleh M (2017). Public stigma toward mental illness in Jordan: a cross-sectional survey of family members of individuals with schizophrenia, depression, and anxiety. J PsychoSoc Nurs Ment Health Serv.

[37] Barakat M (2023). Evaluation of knowledge, experiences, and fear toward prescribing and dispensing corticosteroids among Egyptian healthcare professionals: a cross-sectional study. Saudi Pharm J.

[38] Engels F (2018). Pharmacology education: reflections and challenges. Eur J Pharmacol.

[39] Pathak GN (2023). The Pharmacist’s role in dermatology: patient medication adherence. J Dermatol.

[40] Info. AU. *Universities in Jordan*. 2023 [cited 2023 11/5]; Available from: https://www.alluniversity.info/jordan/.

[41] Basheti IA et al. Primary health care policy and vision for community pharmacy and pharmacists in Jordan. Pharm Pract (Granada). 2020;18(4). 10.18549/PharmPract.2020.4.2184 PMC7732212 33343774

[42] Reddy A (2019). Interruptions in community pharmacies: frequency, sources, and mitigation strategies. Res Social Administrative Pharm.

