# Supplementary material for: An evaluation of pharmacists’ general attitudes, knowledge, and phobias regarding medications that include corticosteroids: a cross-sectional study
Source: J Pharm Health Care Sci. 2024 Feb 1;10:8. doi: 10.1186/s40780-024-00329-x (PMC10832149; doi:10.1186/s40780-024-00329-x)
Supplement: Supplementary file 1 — Supplementary Material 1: Supplementary Material-Study Questionnaire [file 40780_2024_329_MOESM1_ESM.pdf]

An Evaluation of Pharmacists' General Attitudes, Knowledge, And Fears Regarding Medications that Include Corticosteroids: A Cross-Sectional Study.

Section 1. Sociodemographic Data

Age…………………(Years)

Gender

Male

Female

Residential area

Urban

Rural

Pharmacists site of work

Community pharmacy

Hospital pharmacy

Pharmaceutical company

Medical representative

Others

Location of the workplace

North Jordan

Centre of Jordan including the Capital (Amman)

South Jordan

Section 2. Experience with Corticosteroid

Did you ever dispense Corticosteroids for any reason?

Yes

No

Maybe

What was the most common dosage form you ever dispense? (More than one answer is allowed)

Topical (e.g., cream or ointment)

Inhaler or nebulizer

Tablets

Injection

Drops (e.g., eye drops)

What was the main indication for corticosteroid dispensing? (more than one answer is allowed)

Respiratory disease (e.g., Asthma, COPD)

COVID-19

Dermatological disease (e.g., eczema)

Joint or Rheumatological diseases

GIT immunological diseases (e.g., Crohn's disease, ulcerative colitis)

Systemic immunological disorders (e.g., Multiple Sclerosis)

Others

Did you ever experience any of the following side effects with your patients (more than one answer is allowed)?

Increased appetite – potentially leading to weight gain

Acne

Thinned skin that bruises easily

Increased risk of infections

Mood changes, mood swings, and depression

Diabetes

High blood pressure

Osteoporosis (weak and brittle bones)

Others

Section 3. Knowledge about Corticosteroids

Choose the proper option from the following statements.

**Statement**	Yes	No	Not sure
Corticosteroids, often known as steroids, are anti-inflammatory medicine.			
Corticosteroids are man-made hormones normally produced by the adrenal glands.			
Corticosteroids are mainly used to induce inflammation and suppress the immune system.			
Corticosteroids are used to treat various health conditions (e.g., asthma, eczema, COVID-19..etc.)			
Prolonged steroid treatment at low doses – particularly with steroid tablets – can cause problems in some people.			
The used dose needs to be reduced slowly over a few weeks or months before stopping Corticosteroids if you have been taking them for a long time.			
Corticosteroids can cause weight gain			
Corticosteroids can cause skin thinking that bruises easily			
Corticosteroids can cause increased risk of infections			
Corticosteroids can't cause mood changes			
Corticosteroids can cause high blood glucose			

Section 4. Fears towards Corticosteroids prescription

Choose the level of agreement with the following statements.

Statement	Strongly agree	Agree	Neutral	Disagree	Strongly Disagree
Usually, the patients fear of the weight gains due to Corticosteroids use					
Usually, the patients fear of the possible increase in blood sugar due to Corticosteroids use					
Usually, the patients fear of the possible increase in blood pressure due to Corticosteroids use					
Usually, the patients fear of the possible osteoporosis due to Corticosteroids use					
Usually the patients fear of body-dependence or addiction to Corticosteroids and can't live without it					
Usually, the patients fear form oral and injectable Corticosteroids more than the topical products					
Usually, the patients fear of developing adrenal insufficiency after stopping the Corticosteroids					
Usually, the patients fear of social refusal if they know about my Corticosteroids use					
Usually, the patients fear form the possible depression of mood swings due to Corticosteroids use					
Usually the patients fear of possible unknown/untreatable side effects					
